# Clinical efficacy and prognostic evaluation of endovascular treatment in acute basilar artery occlusion

**DOI:** 10.3389/fneur.2026.1597496

**Published:** 2026-04-08

**Authors:** Shu Xu, Zihe Jiang, Yin Zhou, Wenjun Zhang, Yan Zhang

**Affiliations:** 1 Department of Neurology, The Affiliated Zhangjiagang Hospital of Soochow University, Suzhou, China; 2 Department of Rheumatology and Immunology, The Affiliated Zhangjiagang Hospital of Soochow University, Suzhou, China

**Keywords:** acute basilar artery occlusion, best medicalmanagement, complications, endovascular treatment, functional recovery, safety

## Abstract

**Objective:**

To compare the efficacy and prognosis of endovascular treatment (EVT) versus best medical management (BMM) in acute basilar artery occlusion (aBAO).

**Methods:**

We retrospectively analyzed 120 aBAO patients treated at the Affiliated Zhangjiagang Hospital of Soochow University (Jan 2021–Dec 2024), with 60 patients in each group. Baseline characteristics were comparable. Functional recovery was assessed by mRS and EQ-5D at days 0, 30, and 90. Safety outcomes included symptomatic intracranial hemorrhage, 90-day mortality, and functional deterioration. Neurological function and imaging were evaluated using NIHSS and PC-ASPECTS.

**Results:**

EVT showed significantly better mRS at 30 and 90 days (3.0 ± 1.3, 2.5 ± 1.2 vs. 3.8 ± 1.4, 3.3 ± 1.5; *p* = 0.042, 0.025) and higher EQ5D scores (57.6 ± 19.8, 78.8 ± 20.3 vs. 51.5 ± 18.6, 69.6 ± 21.2, *p* = 0.038, 0.015). NIHSS scores were lower in EVT at all time points (*p* < 0.001), PC-ASPECTS higher (7.8 ± 1.2 vs. 6.4 ± 1.5; *p* = 0.001), and basilar artery recanalization rate greater (85% vs. 60%; *p* = 0.002). Safety outcomes showed no significant differences: symptomatic hemorrhage (10% vs. 15%; *p* = 0.45), 90-day mortality (12% vs. 18%; *p* = 0.38), functional deterioration (8% vs. 14%; *p* = 0.32).

**Conclusion:**

EVT significantly improves functional recovery and neurological outcomes in aBAO and demonstrates higher recanalization rates, with comparable safety to BMM, supporting EVT as the preferred treatment option.

## Introduction

Acute basilar artery occlusion (aBAO) is a rare yet highly devastating form of acute cerebrovascular occlusion, accounting for approximately 1% of all strokes (1–3). Due to its extremely high mortality and disability rates, most patients experience severe post-onset functional impairments. Current treatment modalities for aBAO primarily include intravenous and intra-arterial thrombolysis, mechanical thrombectomy, and bridging therapy, with the latter three collectively referred to as endovascular treatment (EVT) (4, 5). In recent years, several clinical trials have confirmed the safety and efficacy of EVT for the treatment of anterior circulation large-vessel occlusions (6, 7).

However, patients with basilar artery occlusion differ significantly from those with anterior circulation stroke in terms of vascular anatomy, clinical presentation, and severity of neurological deficits (8, 9). Therefore, although EVT has gained widespread acceptance for the treatment of anterior circulation occlusions, its efficacy in patients with posterior circulation aBAO remains a subject of debate (10, 11). Multiple large-scale, multicenter randomized clinical trials (RCTs) on posterior circulation aBAO, such as the BEST, BASICS, and BASILAR studies, have explored EVT efficacy (12, 13). However, the results of these studies have been inconclusive. In the BEST and BASICS trials, no significant differences in favorable outcomes at 90 days post-procedure were observed between the EVT and medical management groups, potentially due to suboptimal patient recruitment (14). Moreover, adjustments to the enrollment criteria in the BASICS trial may have further influenced the outcomes (15).

Nonetheless, recent real-world studies and RCTs, such as the BASILAR study and China’s BAOCHE and ATTENTION trials, have provided robust evidence supporting the potential benefits of EVT for the treatment of aBAO (16–18). These studies demonstrated that compared with best medical management, EVT significantly improves favorable outcomes at 90 days and reduces mortality, particularly in severe cases (19). These findings offer new clinical evidence for the treatment of large-vessel occlusions in the posterior circulation and may prompt updates to the clinical guidelines.

Therefore, this study aimed to compare the clinical efficacy and prognosis of EVT and BMM in patients with acute basilar artery occlusion, to provide a basis for further optimization of treatment strategies for aBAO.

## Materials and methods

### Study subjects

Patients with aBAO treated at the Affiliated Zhangjiagang Hospital of Soochow University between January 2021 and December 2024 were retrospectively reviewed. Based on the treatment actually received, patients were categorized into two groups: the EVT group (*n* = 60) and the BMM group (*n* = 60). Inclusion criteria were a confirmed diagnosis of aBAO by imaging, treatment initiation within 6 h of symptom onset, age ≥ 18 years, and provision of informed consent. Exclusion criteria included acute ischemic stroke due to other etiologies, severe comorbidities such as advanced cardiac, hepatic, or renal disease, a history of hemorrhagic stroke or intracerebral hemorrhage, inability to undergo EVT due to significant vascular anatomical abnormalities, pregnancy or lactation, or any condition likely to affect treatment or outcome assessment. Baseline demographic and clinical characteristics were extracted from the hospital electronic medical record system. Variables collected included age, sex, vascular risk factors (hypertension, diabetes mellitus, atrial fibrillation), time from symptom onset to treatment, smoking and alcohol consumption history, total cholesterol, low-density lipoprotein, body mass index, history of previous stroke or coronary artery disease, hemoglobin, and blood glucose levels. All data were systematically screened and verified for accuracy and completeness, minimizing information bias and ensuring reliable retrospective analysis. All participating centers obtained approval from their respective institutional review boards or local ethics committees. The reporting of this study adheres to the Strengthening the Reporting of Observational Studies in Epidemiology (STROBE) guidelines.

### Group and treatment

#### BMM group

Patients in this group received intravenous thrombolysis followed by anticoagulation and antiplatelet therapy. Intravenous thrombolysis was performed using recombinant tissue plasminogen activator (rtPA) at a dosage of 0.9 mg/kg, delivered via intravenous injection within 4.5 h of symptom onset. Following thrombolysis, patients were treated with anticoagulant and antiplatelet therapy. Aspirin was administered orally at a dose of 100 mg once daily.

#### EVT group

The endovascular approach involved mechanical thrombectomy guided by digital subtraction angiography (DSA). A catheter was inserted through the femoral artery and advanced along the aorta to the basilar artery to perform the intervention. The device used for mechanical thrombectomy was the Solitaire™ FR Stent Retriever. During the procedure, the catheter was navigated through the femoral artery and into the basilar artery, where the appropriate interventional device was employed to perform thrombectomy or thrombolysis until vessel recanalization was achieved. Prior to the procedure, imaging studies were conducted to confirm the extent and location of the basilar artery occlusion. Anticoagulation was initiated with heparin (5,000 units). Post-procedure, angiography was used to assess vessel patency, and anticoagulants, as well as antiplatelet therapy (aspirin), were continued to prevent reocclusion.

### Assessment parameters

#### Assessment of functional recovery

Modified Rankin Scale (mRS): This scale assesses functional recovery and ranges from 0 (no symptoms) to 5 (severe disabilities). It reflects recovery based on the functional status of the patient, with assessments conducted on days 0, 30, and 90 after treatment. The mRS is widely used in stroke evaluations to measure functional outcomes (20).

EuroQol 5-Dimensions (EQ-5D) Scale: Health-related quality of life (HRQoL) was assessed using the EuroQol 5-Dimensions instrument (EQ-5D) (21). In the present study, the EQ-5D visual analogue scale (VAS) was used to evaluate patients’ overall perceived health status. The VAS records self-rated health on a vertical scale ranging from 0 (worst imaginable health state) to 100 (best imaginable health state). Assessments were conducted at baseline (day 0) and at 30 and 90 days after treatment.

#### Assessment of neurological function and imaging

National Institutes of Health Stroke Scale (NIHSS): This scale evaluates neurological deficits across various functions, including consciousness, language, and motor skills, with scores ranging from 0 (no deficits) to 42 (severe deficits). Assessments were conducted at baseline (day 0) and at 24 h, 30 days, and 90 days post-treatment. As a key tool in stroke evaluation, the NIHSS quantifies the degree of neurological impairment, aiding in prognosis prediction and treatment decisions (22). Posterior Circulation Acute Stroke Prognosis Early CT Score (PC-ASPECTS): This imaging-based assessment quantifies the volume and severity of ischemic stroke (23). The PC-ASPECTS score ranges from 0 to 10, with higher scores indicating smaller infarct volumes and a better prognosis. The assessment was conducted at 24 h post-treatment.

Basilar Artery Recanalization Rate (BATMAN): This metric evaluates the recanalization of the basilar artery within 24 h hours post-treatment, reflecting the success of the intervention (24). The rate was expressed as the percentage of patients with a patent basilar artery based on imaging studies.

#### Safety and complications monitoring

Incidence of Symptomatic Intracranial Hemorrhage (sICH): This monitored the occurrence of intracranial hemorrhage within 3 days post-treatment, with a focus on symptomatic cases. The proportion of patients who developed sICH was recorded to assess treatment safety.

90-Day Mortality Rate: This metric tracked the mortality rate within 90 days post-treatment to evaluate long-term survival.

Incidence of Functional Deterioration: This monitored the proportion of patients whose functional status worsened within 3 days post-treatment to assess the impact of treatment on patient outcomes.

### Statistical analysis

Data analysis was conducted using SPSS version 26.0. Continuous variables, such as age, total cholesterol, body mass index (BMI), hemoglobin, blood glucose, and time from symptom onset to treatment, were presented as mean ± standard deviation (Mean ± SD). Independent sample t-tests were used for comparisons between groups. For continuous variables that did not follow a normal distribution, data were expressed as median and interquartile range (IQR), and comparisons between groups were performed using the Mann–Whitney U test. Categorical variables, such as sex, history of hypertension, diabetes, atrial fibrillation, and intravenous thrombolysis, were presented as frequencies and percentages. Group differences were tested using the chi-square test or Fisher’s exact test, as appropriate. For functional recovery measures assessed at multiple time points, such as mRS, EQ-5D, and NIHSS scores, as well as imaging-based metrics like PC-ASPECTS score and basilar artery recanalization rate, repeated measures analysis of variance (ANOVA) or mixed-effects models were employed to evaluate the interaction effects between time and group. All statistical tests were two-sided, and a *p*-value lower than 0.05 was considered statistically significant.

## Results

### Comparison of baseline characteristics between the two groups

In this study, 120 patients with aBAO were randomly assigned to the EVT and BMM groups. The baseline characteristics of patients in both groups were comparable, with no statistically significant differences (Table 1). Specifically, the mean age of patients in the EVT group was 65.2 ± 10.3 years, compared to 64.8 ± 11.2 years in the BMM group (*p* = 0.823). The sex distribution was similar, with 63.3% of male patients in the EVT group and 60.0% in the BMM group (*p* = 0.735). There were no significant differences between the two groups regarding history of hypertension (*p* = 0.683), diabetes (*p* = 0.688), atrial fibrillation (*p* = 0.674), or prior intravenous thrombolysis (*p* = 0.688). Additionally, other baseline characteristics such as time from symptom onset to treatment, smoking history, alcohol consumption, total cholesterol, low-density lipoprotein, BMI, history of previous stroke, coronary artery disease, hemoglobin levels, and blood glucose levels did not differ significantly between the two groups (*p* > 0.05). These findings indicate that the clinical characteristics of the patients at baseline were balanced between the two groups, facilitating a fair comparison of treatment efficacy and safety in subsequent analyses.

**Table 1:** Comparison of baseline characteristics between the two groups.

Baseline information		EVT group (*n* = 60)	BMM group (*n* = 60)	*P-*value
Age		65.2 ± 10.3	64.8 ± 11.2	0.823
Sex	Male	38 (63.3%)	36 (60.0%)	0.735
	Female	22(36.7%)	24(40.0%)	0.625
Hypertension	Yes	40 (66.7%)	42 (70.0%)	0.683
	No	20(33.3%)	18(30%)	*0.3317*
Diabetes	Yes	15 (25.0%)	17 (28.3%)	0.688
	No	45(75.0%)	43(71.7%)	0.312
Atrial fibrillation	Yes	12 (20.0%)	14 (23.3%)	0.674
	No	48(80.0%)	46(76.7%)	0.326
Intravenous thrombolytic therapy		18 (30.0%)	20 (33.3%)	0.688
Time from onset to treatment (h)		6.2 ± 1.5	6.4 ± 1.7	0.548
Smoking history	Yes	25 (41.7%)	27 (45.0%)	0.724
	No	35(58.3%)	33(55.0%)	0.276
Alcohol consumption history	Yes	20 (33.3%)	22 (36.7%)	0.743
	No	40(66.7%)	38(63.3%)	0.257
Total cholesterol (mmol/L)		5.2 ± 1.1	5.1 ± 1.2	0.685
LDL (mmol/L)		3.3 ± 0.9	3.2 ± 1.0	0.721
BMI (kg/m^2^)		25.8 ± 3.4	26.1 ± 3.6	0.638
Stroke history	Yes	10 (16.7%)	12 (20.0%)	0.648
	No	50 (83.3%)	48 (80.0%)	0.352
Coronary heart disease	Yes	14 (23.3%)	13 (21.7%)	0.823
	No	46 (76.7%)	47 (78.3%)	0.177
Hemoglobin (g/L)		140 ± 15	138 ± 14	0.512
Blood glucose (mmol/L)		6.8 ± 2.0	6.7 ± 2.1	0.784

BMI, body mass index; BMM, best medical management; EVT, endovascular treatment; LDL, low-density lipoprotein.

### Comparison of functional recovery between the two groups

Functional recovery of patients in the EVT group was compared with that in the BMM group at multiple time points. The results indicated that EVT was superior to BMM in terms of functional recovery (Table 2). Specifically, there were no significant differences in mRS scores between the two groups at baseline (Day 0), with the EVT group having an average score of 4.5 ± 1.0 and the BMM group having an average score of 4.6 ± 1.1 (*p* = 0.645). However, over time, the mRS scores improved significantly in the EVT group. At 30 days, the average mRS score in the EVT group was 3.0 ± 1.3, compared to 3.8 ± 1.4 in the BMM group, with a statistically significant difference (*p* = 0.042). By 90 days, the average score for the EVT group further decreased to 2.5 ± 1.2, whereas that for the BMM group was 3.3 ± 1.5. The difference was still significant (*p* = 0.025).

**Table 2:** Comparison of mRS and EQ-5D scores between the two groups.

Assessment indicator	Time	EVT group (*n* = 60)	BMM group (*n* = 60)	*P-*value
mRS score (Mean ± SD)	0 days	4.5 ± 1.0	4.6 ± 1.1	0.645
	30 days	3.0 ± 1.3	3.8 ± 1.4	0.042
	90 days	2.5 ± 1.2	3.3 ± 1.5	0.025
EQ-5D score (Mean ± SD)	0 days	42.4 ± 18.5	41.4 ± 17.9	0.732
	30 days	57.6 ± 19.8	51.5 ± 18.6	0.038
	90 days	78.8 ± 20.3	69.6 ± 21.1	0.015

BMM, best medical management; EVT, endovascular treatment; EQ-5D, EuroQol 5-dimensions; mRS, Modified Rankin Scale.

In terms of health-related quality of life, the EQ-5D scores showed no significant difference between the two groups at Day 0, with the EVT group scoring 42.4 ± 18.5 and the BMM group scoring 41.4 ± 17.9 (*p* = 0.732). However, as treatment progressed, the EQ-5D scores for the EVT group gradually increased. At 30 days, the EVT group had an average score of 57.6 ± 19.8, which was significantly higher than that for the BMM group (51.5 ± 18.6; *p* = 0.038). By 90 days, the score for the EVT group reached 78.8 ± 20.3 and that for the BMM group 69.6 ± 21.1, with the level of statistical significance of the difference becoming even more pronounced (*p* = 0.015).

These findings indicate that EVT provides a substantial advantage in improving long-term functional recovery and health-related quality of life for patients.

### Safety and complication comparison

In the comparison of safety and complications, the results showed differences in symptomatic intracranial hemorrhage, 90-day mortality, and functional deterioration rates between the two groups; however, none reached statistical significance. Specifically, within 3 days post-treatment, the incidence of symptomatic intracranial hemorrhage was 10% (6/60) in the EVT group and 15% (9/60) in the BMM group, with no statistically significant difference between the groups (*p* = 0.45). At 90 days, the mortality rate was 12% (7/60) in the EVT group and 18% (11/60) in the BMM group, again showing no significant difference (*p* = 0.38). Additionally, the incidence of functional deterioration within 3 days was 8% (5/60) in the EVT group and 13% (8/60) in the BMM group; however, the difference was not statistically significant (*p* = 0.32). Overall, although the EVT group exhibited a relatively lower incidence of adverse events across these safety indicators, these differences did not achieve statistical significance, indicating that the overall safety and complication profiles of the two groups were similar (Table 3).

**Table 3:** Comparison of safety and complications under different treatments.

Safety and complications	EVT group (*n* = 60)	BMM group (*n* = 60)	*P-*value
Symptomatic intracranial hemorrhage (%)	10% (6/60)	15% (9/60)	0.45
90-day mortality (%)	12% (7/60)	18% (11/60)	0.38
Functional deterioration within 3 days (%)	8% (5/60)	13% (8/60)	0.32

BMM, best medical management; EVT, endovascular treatment.

### Comparison of neurological function and imaging assessments between the two groups

In terms of neurological function and imaging assessments, significant differences were observed between the EVT and BMM groups (Table 4). Initially, the NIHSS scores, which assess neurological deficits, were comparable between the two groups before treatment (Day 0), with the EVT group scoring 18.2 ± 4.5 and the BMM group scoring 17.8 ± 4.2 (*p* = 0.68). However, within 24 h after treatment, the EVT group showed a significant reduction in NIHSS scores to 13.0 ± 4.3, compared to 15.5 ± 4.1 in the BMM group (*p* = 0.015). At 30 and 90 days post-treatment, the EVT group NIHSS scores further decreased to 8.5 ± 3.8 and 6.0 ± 3.5, respectively, while the BMM group scored 12.0 ± 4.0 and 9.5 ± 3.7 at the same time points, with the differences reaching statistical significance (*p* < 0.001). These findings indicate a more pronounced neurological recovery in the EVT group.

**Table 4:** Comparison of neurological function recovery and imaging assessments between the two groups.

Assessment	Time	EVT group (*n* = 60)	BMM group (*n* = 60)	*P-*value
NIHSS score (Mean ± SD)	Day 0	18.2 ± 4.5	17.8 ± 4.2	0.68
	24 h post-treatment	13.0 ± 4.3	15.5 ± 4.1	0.015
	Day 30 post-treatment	8.5 ± 3.8	12.0 ± 4.0	<0.001
	Day 90 post-treatment	6.0 ± 3.5	9.5 ± 3.7	<0.001
PC-ASPECTS score (Mean ± SD)	24 h post-treatment	7.8 ± 1.2	6.4 ± 1.5	0.001
Basilar artery recanalization rate (%)	24 h post-treatment	85% (51/60)	60% (36/60)	0.002

BMM, best medical management; EVT, endovascular treatment; NIHSS, National Institutes of Health Stroke Scale; PC-ASPECTS, posterior circulation acute stroke prognosis early CT score.

Regarding imaging assessments, the EVT group demonstrated significantly higher PC-ASPECTS scores 24 h after treatment, with an average score of 7.8 ± 1.2 compared to 6.4 ± 1.5 in the BMM group (*p* = 0.001). This finding suggests a smaller infarct volume and less severe brain damage in the EVT group. Additionally, the recanalization rate of the basilar artery within 24 h was significantly higher in the EVT group at 85% (51/60), compared with 60% (36/60) in the BMM group (*p* = 0.002). Overall, these results indicate that the EVT group outperformed the BMM group in both neurological recovery and imaging-based assessments.

## Discussion

The optimal treatment strategy for aBAO remains an important and evolving issue in stroke management (25). While EVT has become the standard treatment for anterior circulation large-vessel occlusions, its efficacy in posterior circulation stroke has historically been less certain (26). Early randomized controlled trials, including BEST and BASICS, did not demonstrate a clear functional benefit of EVT over medical therapy in patients with aBAO (27).

Recently, accumulating evidence has supported the potential benefit of EVT in patients with aBAO. The BASILAR study, a large prospective registry, reported that EVT was associated with improved functional outcomes and reduced one-year mortality in real-world clinical practice (28). Subsequently, the ATTENTION trial demonstrated that EVT significantly improved functional outcomes compared with BMM in patients treated within an early therapeutic window (29). In addition, the BAOCHE trial further extended the therapeutic window and showed that EVT remained beneficial in patients treated 6–24 h after symptom onset when appropriate imaging selection criteria were applied (10). Together, these studies have strengthened the evidence supporting EVT as an effective treatment strategy for selected patients with aBAO. Consistent with these recent findings, the results of the present study demonstrated that patients treated with EVT achieved significantly better functional outcomes than those receiving BMM, particularly with respect to mRS and EQ-5D scores at 30 and 90 days. These findings suggest that timely endovascular reperfusion may play an important role in improving neurological recovery and overall functional status in patients with aBAO. Therefore, our results provide additional real-world evidence supporting the clinical value of EVT in the management of aBAO.

The results of this study demonstrated that the EVT group exhibited significantly better functional recovery than the BMM group, particularly in terms of mRS and EQ-5D scores at 30 and 90 days. These findings align with those of recent studies, such as ATTENTION and BAOCHE, which reported that the EVT group had a higher rate of favorable functional outcomes at 90 days (30). This improvement may be attributed to the ability of EVT to rapidly restore blood flow in the basilar artery and reduce the duration of ischemia, thereby mitigating neural damage and facilitating long-term recovery. Additionally, Abdalkader et al. (31) showed that the EVT group had a higher likelihood of achieving an mRS of 0–3 at 90 days, but also a higher incidence of symptomatic intracranial hemorrhage, while demonstrating lower mortality. Furthermore, Chen et al. (32) noted that EVT was associated with better functional recovery and lower mortality risk in patients with BAO, although they also showed a higher risk of symptomatic intracranial hemorrhage. These findings are consistent with the results of the present study, further supporting the use of EVT as an effective treatment for aBAO.

Although the EVT group demonstrated significant advantages in functional recovery, there were no statistically significant differences between the EVT and BMM groups in terms of safety indicators such as incidence of symptomatic intracranial hemorrhage, 90-day mortality, and functional deterioration rates. Shuo et al. (33) reported that in patients with atrial fibrillation complicated by basilar artery occlusion, there were no significant differences in symptomatic intracranial hemorrhage or mortality between the EVT and BMM groups at 90 days. Similarly, Chuanhui et al. (34) found that endovascular treatment did not significantly affect 90-day mortality, 24-h symptomatic intracranial hemorrhage rates, or the number of procedure-related complications. These results suggest that, in terms of safety, EVT does not significantly increase the risk of complications.

Regarding neurological function recovery, the EVT group demonstrated superior performance compared to the BMM group, as reflected by the significantly lower NIHSS scores at 24, 30, and 90 days post-treatment. This indicates that EVT is more effective in mitigating neurological deficits, likely because of its ability to rapidly restore blood flow and reduce ischemic damage. Yoshimoto et al. (8) found that in patients with severe neurological deficits due to aBAO, those treated with EVT were more likely to walk independently compared to those receiving BMM, while patients with mild neurological deficits that received either treatment showed similar independence rates. Additionally, the EVT group exhibited significantly higher PC-ASPECTS scores and basilar artery recanalization rates on imaging assessment, further supporting the superiority of EVT in reducing infarct volume and improving vascular patency. Therefore, EVT has significant clinical value as a treatment option for aBAO, with notable advantages in terms of neurological recovery and vascular recanalization.

## Conclusion and limitations

The findings of this study provide additional evidence supporting the use of EVT in the management of aBAO. Although some safety outcomes did not reach statistical significance, EVT demonstrated advantages in functional recovery, neurological outcomes, and imaging assessments. These results suggest that EVT may be a valuable therapeutic option for patients with aBAO and may provide useful insights for optimizing clinical management strategies.

However, several limitations should be acknowledged. First, the relatively small sample size may have reduced the statistical power of the analysis, particularly in detecting subtle differences between groups and identifying rare adverse events. Second, this was a single-center study, and the patient population was derived from a single institution. Therefore, regional differences in medical resources, treatment strategies, and patient characteristics may limit the generalizability of the findings. In addition, the retrospective study design means that treatment allocation was based on the therapy actually received rather than randomized assignment, which may introduce selection bias and potential confounding. Although baseline characteristics between the two groups were comparable, advanced statistical adjustment methods, such as multivariable regression or propensity score matching, were not applied. Consequently, residual confounding cannot be completely excluded. Furthermore, the follow-up period was limited to 90 days. While this time point is widely used as a standard endpoint in stroke studies for functional outcome assessment, it may not fully capture long-term functional recovery, cognitive outcomes, or sustained improvements in health-related quality of life. Longer-term follow-up is therefore warranted to further evaluate the long-term efficacy and safety of EVT in patients with aBAO. Finally, although no significant differences in safety outcomes were observed between the two groups, the limited sample size and study design may have restricted the statistical power to detect rare adverse events. Larger, multicenter, prospective studies are needed to further validate the efficacy and safety of EVT in the treatment of aBAO. In summary, while the findings of this study are encouraging, further large-scale, multicenter randomized controlled trials are required to confirm these results.

## Data Availability

The datasets presented in this study can be found in online repositories. The names of the repository/repositories and accession number(s) can be found in the article/supplementary material.
